# Biologic relief without the price tag: the promise of omlcylo in atopic asthma and chronic spontaneous urticaria

**DOI:** 10.1097/MS9.0000000000003786

**Published:** 2025-08-28

**Authors:** Anna Mumtaz, Kamran Shahid, Maliha Khalid, Aminath Waafira

**Affiliations:** a Karachi Medical and Dental College, Karachi, Pakistan; b Shaikh Khalifa Bin Zayed Al-Nahyan Medical and Dental College, Lahore, Pakistan; c Jinnah Sindh Medical University, Karachi, Pakistan; d The Maldives National University, Malé, Maldives

**Keywords:** atopic asthma, chronic spontaneous urticaria, omalizumab biosimilar, omlyclo

## Abstract

Omlyclo (omalizumab-igec), the first FDA-approved biosimilar to omalizumab (Xolair) in the United States, was approved on 7 March 2025, for multiple indications including atopic asthma, chronic rhinosinusitis with nasal polyps, IgE-mediated food allergy, and chronic spontaneous urticaria (CSU). Functioning as an anti-IgE monoclonal antibody, Omlyclo binds free IgE, preventing its interaction with FcεRI receptors on mast cells and basophils, thereby reducing inflammatory responses. Clinical trials, including a pivotal global Phase 3 study in CSU, demonstrated comparable efficacy to Xolair with a significantly reduced cost, enhancing accessibility for patients. While generally well tolerated, reported side effects include pruritus, vertigo, and gastrointestinal disturbances, with rare concerns regarding malignancy risk requiring further long-term evaluation. The availability of this cost-effective biosimilar marks an important milestone in expanding biologic treatment access for patients with allergic asthma and CSU.

*To the Editor*,

On March 7, The U.S. Food and Drug Administration (FDA) approved Omlyclo (omalizumab-igec) for the treatment of asthma, chronic rhinosinusitis with nasal polyps, IgE-mediated food allergy, and chronic spontaneous urticaria (CSU), being a particular biosimilar to Xolair and the first biosimilar to omalizumab in the United States^[1]^.

Omlyclo (omalizumab-igec) is an anti-IgE antibody used to treat a variety of allergy disorders and moderate-to-severe persistent asthma in adults and children with positive allergy testing and poor control with inhaled corticosteroids. It may also be used as an add-on treatment for ongoing rhinosinusitis with nasal polyps (CRSwNP) in individuals who are resistant to nasal corticosteroids; it additionally minimizes allergic reactions, including anaphylaxis, in patients with IgE-mediated food allergies when administered in conjunction with allergen avoidance and significantly approved for chronic spontaneous urticaria (CSU) in adults who remain symptomatic despite H1 antihistamine treatment^[2]^.

Allergic asthma and CSU are IgE-mediated diseases that cause persistent inflammation. In asthma, IgE causes bronchoconstriction and airway hyperresponsiveness; in contrast, CSU causes mast cell degranulation, resulting in recurring hives and angioedema characterized by redness and swelling^[3]^. Despite using high-dose antihistamines and inhaled corticosteroids (ICS), many asthma patients still have symptoms, suggesting the need for more potent therapies. Standard medications are insufficient for about 50% of individuals with severe asthma, underscoring the need for tailored biologic therapies. Omalizumab, an anti-IgE antibiotic, has demonstrated noteworthy efficacy in patients with atopic asthma^[4]^.

Omlyclo, identical to omalizumab, works by binding free IgE and inhibiting its attachment to FcεRI receptors on mast cells and basophils–suppressing inflammatory cascades, thus lessening exacerbations and symptoms^[4]^. Conveniently, it can be administered subcutaneously every 2-4 weeks instead of orally every day^[5]^.

This FDA approval follows positive outcomes observed through comprehensive clinical evidence, most notable among them being a global phase 3 trial involving 690 patients with CSU. The results of the trial demonstrated similar efficacy to the comparator Xolair, while being available for a fraction of the cost, highlighting the significance of such a development^[1]^.

Generally, the drug was well-tolerated, and serious adverse effects were rare, however, side effects observed during trials ranged from arthritis, pruritus, and vertigo to gastroenteritis and pharyngitis. The drug has been strictly contraindicated in cases of patient hypersensitivity to Omlyclo or its ingredients. Furthermore, some clinical studies report increased development of various types of malignancies, with long-term risks still being unknown^[1]^.

Omlyclo gives a promising, affordable alternative to existing treatments, proving to be quite effective in clinical trials. It has the capability of changing the approach toward the treatment of atopic asthma and CSU, however further research is needed to address its long-term concerns as well as acquire more detailed information about its administration and effectiveness. Our work is in line with the TITAN Guidelines on the need for transparency in AI use in healthcare^[6]^.

## Data Availability

No data were generated for this manuscript.

## Peer and provenance statement

This article was not commissioned and was externally peer-reviewed.

## References

[1] FDA approves omlyclo (omalizumab-igec), an interchangeable biosimilar to xolair [Internet]. Drugs.com. [cited 2025 May 13]. https://www.drugs.com/newdrugs/fda-approves-omlyclo-omalizumab-igec-interchangeable-biosimilar-xolair-6473.html

[2] 761399s000lbl.pdf [Internet]. [cited 2025 May 13]. https://www.accessdata.fda.gov/drugsatfda_docs/label/2025/761399s000lbl.pdf

[3] Venkatesan P. 2023 GINA report for asthma. Lancet Respir Med 2023;11:589. 37302397 10.1016/S2213-2600(23)00230-8

[4] Treating severe allergic asthma with anti-IgE monoclonal antibody (omalizumab): a review - PMC [Internet]. [cited 2025 May 14]. https://pmc.ncbi.nlm.nih.gov/articles/PMC4113133/10.1186/2049-6958-9-23 PMC4113133 24735949

[5] Brás R Costa C Limão R. Omalizumab in Chronic Spontaneous Urticaria (CSU): real-life experience in dose/interval adjustments and treatment discontinuation. J Allergy Clin Immunol Pract 2023;11:2392–402. 36720390 10.1016/j.jaip.2023.01.022

[6] Agha RA Mathew G Rashid R. Transparency in the reporting of Artificial INtelligence – the TITAN guideline. Prem J Sci 2025;10:100082.

